# Supplementary material for: Effects of vaccination against COVID-19 on the emotional health of older adults
Source: F1000Res. 2023 May 16;11:868. Originally published 2022 Jul 29. [Version 2] doi: 10.12688/f1000research.123395.2 (PMC11362718; doi:10.12688/f1000research.123395.2)
Supplement: Peer review history

# Reviewer report: Reviewer response for version 2

**DOI:** 10.5256/f1000research.146973.r173813

**Published:** 2024-09-14

**Authors:** Javier Mariani

**Affiliations:** 1 Department of Cardiology, Hospital El Cruce Néstor C. Kirchner, Buenos Aires, Argentina

The authors addressed appropriately almost all comments. However, the conclusions (both in the abstract and in the main text) remain causal:

- "Two doses of COVID-19 vaccination in older adults improves their perception of COVID-19 infection consequences" (abstract).
- "Vaccination against COVID-19 with two doses in older adults reduces fear and anxiety about COVID-19, compared to those who were unvaccinated or had only one dose" (main text). 

This should be reconsidered since it is not possible to affirm that the perceptions are caused by the vaccine's number of doses. Instead, the study found an association between the number of doses received and the perceptions of fear and anxiety. Also, the second sentence of the conclusion: "However, we observed no effect in general anxiety and general depression, nor in those who only had one vaccine dose compared to those who were unvaccinated", should be reconsidered, since the study did not find an association.

Is the work clearly and accurately presented and does it cite the current literature?

Yes

If applicable, is the statistical analysis and its interpretation appropriate?

Partly

Are all the source data underlying the results available to ensure full reproducibility?

Yes

Is the study design appropriate and is the work technically sound?

Partly

Are the conclusions drawn adequately supported by the results?

No

Are sufficient details of methods and analysis provided to allow replication by others?

Yes

Reviewer Expertise:

Cardiology - COVID.

I confirm that I have read this submission and believe that I have an appropriate level of expertise to confirm that it is of an acceptable scientific standard, however I have significant reservations, as outlined above.

# Reviewer report: Reviewer response for version 2

**DOI:** 10.5256/f1000research.146973.r206873

**Published:** 2024-08-29

**Authors:** Aftab Ahmad

**Affiliations:** 1 Department of Community Medicine, Teerthanker Mahaveer Medical College and Research Center, Moradabad, India

A good research article which has been done in a scientifically sound manner, The authors have written the discussion and conclusion very well, without overarching on the study findings. However, the topic has lost its relevance in the current scenario. Nonetheless, this is an importance piece of research and warrants indexing for academic purposes.

Is the work clearly and accurately presented and does it cite the current literature?

Yes

If applicable, is the statistical analysis and its interpretation appropriate?

Yes

Are all the source data underlying the results available to ensure full reproducibility?

Yes

Is the study design appropriate and is the work technically sound?

Yes

Are the conclusions drawn adequately supported by the results?

Yes

Are sufficient details of methods and analysis provided to allow replication by others?

Yes

Reviewer Expertise:

Mental Health, Epidemiology

I confirm that I have read this submission and believe that I have an appropriate level of expertise to confirm that it is of an acceptable scientific standard.

# Reviewer report: Reviewer response for version 1

**DOI:** 10.5256/f1000research.135497.r159978

**Published:** 2023-01-30

**Authors:** Javier Mariani

**Affiliations:** 1 Department of Cardiology, Hospital El Cruce Néstor C. Kirchner, Buenos Aires, Argentina

The authors report the results of an observational study assessing the association between vaccination status and mental health outcome variables at two-time points.

 Overall, the study is very interesting and the addressed theme is relevant. However, the manuscript has several issues that should be reconsidered.

- The study aimed to evaluate the effects of vaccination on mental health outcomes, and this study design is not the most appropriate to accomplish that objective. The study evaluated the association between mental health measures and vaccination status (indeed participants were already vaccinated -the exposure- when outcomes were measured. This should be recognized and the paper reformulated consequently; that is, there are several causal relationship sentences, for example: “High fear and anxiety about COVID-19 significantly decreased in those who had two doses of the COVID-19 vaccine than those who were unvaccinated” (Discussion, second paragraph), that should be reconsidered.
- Table 1 does not have measures of association between groups and the variables in the table. Please, include p values or uncertainty measures that help to understand the distribution of baseline characteristics among study groups.
- The analysis of the outcome as a dichotomic variable could affect the power to detect differences. As an example, the medians and IQR for general anxiety and depression are 0 for almost all groups (only the group one-dose has a general anxiety 75 ^th^ percentile of 1); the dichotomization at 2 and 3 points, respectively, leaves most participants as 0. Also, the presentation medians and IQR in the results tables, do not represent the analyses, if this analysis strategy will be maintained, the tables should show the number and proportion of participants with the outcome. Other strategies, such as Poisson regression models (or some variant for zero-inflated data) could have some advantages.
- There are no p values for association tests reported, please add this information to figure 3 and tables 2 and 3.
- The unvaccinated group was small, which could affect the study's power to detect between-group differences.
- Please, include comparisons between one-dose and two-doses groups in tables 2 and 3, and in figure 3.

Minor comment

- Check the spelling.

Is the work clearly and accurately presented and does it cite the current literature?

Yes

If applicable, is the statistical analysis and its interpretation appropriate?

Partly

Are all the source data underlying the results available to ensure full reproducibility?

Yes

Is the study design appropriate and is the work technically sound?

Partly

Are the conclusions drawn adequately supported by the results?

No

Are sufficient details of methods and analysis provided to allow replication by others?

Yes

Reviewer Expertise:

Cardiology - COVID.

I confirm that I have read this submission and believe that I have an appropriate level of expertise to confirm that it is of an acceptable scientific standard, however I have significant reservations, as outlined above.

# Response

**Published:** 2023-04-12

**Authors:** Christoper A. Alarcon-Ruiz

**Affiliations:** Universidad Científica del Sur, Peru

1. “The study aimed to evaluate the effects of vaccination on mental health outcomes, and this study design is not the most appropriate to accomplish that objective. The study evaluated the association between mental health measures and vaccination status (indeed participants were already vaccinated -the exposure- when outcomes were measured. This should be recognized and the paper reformulated consequently; that is, there are several causal relationship sentences, for example: “High fear and anxiety about COVID-19 significantly decreased in those who had two doses of the COVID-19 vaccine than those who were unvaccinated” (Discussion, second paragraph), that should be reconsidered.”

 Response: Thanks for the suggestion, we corrected the phrasing of our results, according to the study design.

 2. "Table 1 does not have measures of association between groups and the variables in the table. Please, include p values or uncertainty measures that help to understand the distribution of baseline characteristics among study groups.”

 Response: We added p values in Table 1 and described them in the results section.

 3.“The analysis of the outcome as a dichotomic variable could affect the power to detect differences. As an example, the medians and IQR for general anxiety and depression are 0 for almost all groups (only the group one-dose has a general anxiety 75th percentile of 1); the dichotomization at 2 and 3 points, respectively, leaves most participants as 0. Also, the presentation medians and IQR in the results tables, do not represent the analyses, if this analysis strategy will be maintained, the tables should show the number and proportion of participants with the outcome. Other strategies, such as Poisson regression models (or some variant for zero-inflated data) could have some advantages.”

 Response: We agree with the suggestions. We excluded the analysis comparing medians and IQR of the outcomes. We are using a validated cut-off for all our outcomes, considering the interpretation of our results, considering them as clinical relevant outcomes for the participants.

 4. “There are no p values for association tests reported, please add this information to figure 3 and tables 2 and 3.”

 Response: Thanks for the suggestion. However, we are reporting 95%CI of the estimates. These values are better to understand the statistical significance of our results.

 5. “The unvaccinated group was small, which could affect the study's power to detect between-group differences.”

 Response: We acknowledge this situation as a limitation in the final paragraph of the Discussion section.

 6. “Please, include comparisons between one-dose and two-doses groups in tables 2 and 3, and in figure 3.”

 Response: Thanks for the suggestions. We added these analysis in tabls 2 and 3, and included on the discussion of our results. The figure 3 already included this comparison.

# Reviewer report: Reviewer response for version 1

**DOI:** 10.5256/f1000research.135497.r146036

**Published:** 2022-08-30

**Authors:** Oscar Rosas-Carrasco

**Affiliations:** 1 Evaluation and intervention Center of Older People, Multi-component exercise Unit, Health department, Ibero-American University, Mexico City, Mexico

The ideal study to demonstrate the effectiveness of vaccination in mental health are intervention studies. However, this follow-up study may provide sufficient evidence to demonstrate the association between improved mental health status in the group that received two vaccines versus those that received no or one dose. Therefore, the knowledge provided by this study is relevant and should be reported.

 In the introduction, the percentages of vaccination with three vaccines are described, in its majority it is commented that they were vaccinated with Pfizer, that the Ministry of Health gave preference to this vaccine for older adults, however, in the methods it is not specified if it was a exclusion criterion having been vaccinated with other types of vaccines. In this case, if they had been selected add this variable as it is confusing. On the other hand, the adverse effects of vaccines occur frequently, if they occur they could have an effect contrary to the reduction of fear, anxiety, etc. due to COVID-19. Could the effects or adverse reactions be quantified? If so, include them as another confounding variable in the methodology.

 It is necessary to deepen in the section because no significant differences were found in terms of depression and anxiety. Perhaps a paragraph should be included on the fact that using general questionnaires for anxiety and depression decreases their sensitivity for the detection of very specific changes such as that of vaccination? Had questionnaires developed (for this work) been used for this specific purpose, a significant change could have been found.

 Another line that can be included in the discussion is that the presentation of depression and anxiety and its change does not only depend on the vaccination against COVID-19, it depends on many factors that can exert a greater weight than the vaccination itself and that for this reason an impact could not be obtained, future studies with greater strength in the operational definition of the variables or with an intervention design could help to clarify these two aspects of mental health.

 The part in the argument that three or four doses could further improve mental health status is well supported.

Is the work clearly and accurately presented and does it cite the current literature?

Yes

If applicable, is the statistical analysis and its interpretation appropriate?

Yes

Are all the source data underlying the results available to ensure full reproducibility?

Yes

Is the study design appropriate and is the work technically sound?

Partly

Are the conclusions drawn adequately supported by the results?

Yes

Are sufficient details of methods and analysis provided to allow replication by others?

Yes

Reviewer Expertise:

Internal Medicine, Geriatric Medicine, clinical research

I confirm that I have read this submission and believe that I have an appropriate level of expertise to confirm that it is of an acceptable scientific standard, however I have significant reservations, as outlined above.

# Response

**Published:** 2023-04-12

**Authors:** Christoper A. Alarcon-Ruiz

**Affiliations:** Universidad Científica del Sur, Peru

1. “The ideal study to demonstrate the effectiveness of vaccination in mental health are intervention studies. However, this follow-up study may provide sufficient evidence to demonstrate the association between improved mental health status in the group that received two vaccines versus those that received no or one dose. Therefore, the knowledge provided by this study is relevant and should be reported.”

 Response: Thanks.

 2. “In the introduction, the percentages of vaccination with three vaccines are described, in its majority it is commented that they were vaccinated with Pfizer, that the Ministry of Health gave preference to this vaccine for older adults, however, in the methods it is not specified if it was a exclusion criterion having been vaccinated with other types of vaccines. In this case, if they had been selected add this variable as it is confusing. On the other hand, the adverse effects of vaccines occur frequently, if they occur they could have an effect contrary to the reduction of fear, anxiety, etc. due to COVID-19. Could the effects or adverse reactions be quantified? If so, include them as another confounding variable in the methodology.”

 Response: We included all participants independently of vaccine preference or the type of received vaccine (We are adding this information in the methods section). In addition, we had the variable vaccine preference in the database, and it was included in the multivariable analysis. However, we didnt collect information about the adverse effects of the vaccines, but we are acknowledging that as a limitation.

 3. “It is necessary to deepen in the section because no significant differences were found in terms of depression and anxiety. Perhaps a paragraph should be included on the fact that using general questionnaires for anxiety and depression decreases their sensitivity for the detection of very specific changes such as that of vaccination? Had questionnaires developed (for this work) been used for this specific purpose, a significant change could have been found.”

 Response: We agree with the reviewer and add the following text to the limitations section:

*“Fifthly, we used short and general questionnaires to measure anxiety and depressive symptoms (i.e. PHQ-2 and GAD-2), which are not specific to vaccination settings. Therefore, it is possible that significant differences would have been found if instruments specifically designed for these settings had been used. Similarly, it is possible that the use of more comprehensive versions measuring depressive and anxiety symptoms, such as the PHQ-9 and GAD-7, which include emotional and somatic indicators, might have increased the variability of the measures and found significant results. However, we believe that using the PHQ-2 and GAD-2 captures the core symptoms of anxiety and depression.”*

 4. “Another line that can be included in the discussion is that the presentation of depression and anxiety and its change does not only depend on the vaccination against COVID-19, it depends on many factors that can exert a greater weight than the vaccination itself and that for this reason an impact could not be obtained, future studies with greater strength in the operational definition of the variables or with an intervention design could help to clarify these two aspects of mental health.”

 Response: We have included this information in the limitations section, as we believe it is an issue not covered in our study:

*“Sixth, it is possible that the change in outcomes such as anxiety or depressive symptoms in older adults was not solely dependent on COVID-19 vaccination. Social determinants of health or other factors may be more influential and not accounted for in our study (i.e., family support, economic status, or quality of life). Therefore, invite other researchers to design future studies with greater methodological rigour, taking into account the considerations mentioned in our limitations.”*
